# The daunting polygenicity of mental illness: making a new map

**DOI:** 10.1098/rstb.2017.0031

**Published:** 2018-01-29

**Authors:** Steven E. Hyman

**Affiliations:** 1 Stanley Center, Broad Institute of MIT and Harvard, 75 Ames Street, Cambridge, MA 02142, USA; 2 Department of Stem Cell and Regenerative Biology, Harvard University, Cambridge, MA, USA

**Keywords:** polygenic, schizophrenia, autism spectrum, brain organoid

## Abstract

An epochal opportunity to elucidate the pathogenic mechanisms of psychiatric disorders has emerged from advances in genomic technology, new computational tools and the growth of international consortia committed to data sharing. The resulting large-scale, unbiased genetic studies have begun to yield new biological insights and with them the hope that a half century of stasis in psychiatric therapeutics will come to an end. Yet a sobering picture is coming into view; it reveals daunting genetic and phenotypic complexity portending enormous challenges for neurobiology. Successful exploitation of results from genetics will require eschewal of long-successful reductionist approaches to investigation of gene function, a commitment to supplanting much research now conducted in model organisms with human biology, and development of new experimental systems and computational models to analyse polygenic causal influences. In short, psychiatric neuroscience must develop a new scientific map to guide investigation through a polygenic *terra incognita*.

This article is part of a discussion meeting issue ‘Of mice and mental health: facilitating dialogue between basic and clinical neuroscientists’.

## 1. Introduction

Research on such disabling psychiatric illnesses as schizophrenia, mood disorders and autism spectrum disorders (ASDs) has been stymied by the complexity of the human brain, its inviolability in life and a dearth of neuropathological clues to pathogenic mechanisms. Even when anatomic stigmata of psychiatric illnesses have been identified, molecular information has not been evident. For example, structural magnetic resonance imaging in schizophrenia demonstrates excessive thinning of prefrontal and temporal cerebral cortex [1]. Post-mortem examination suggests that such pathologic grey matter loss results from a decrement in synaptic spines [2]. However, in contrast to the biochemical neuropathology found in late life neurodegenerative diseases, such as aggregates of Aβ, tau and α-synuclein, the structural abnormalities of schizophrenia do not directly implicate molecular mechanisms. Further, the cognitive and behavioural functions of the human brain most germane to schizophrenia, mood disorders, ASDs, and other early onset psychiatric disorders have proven particularly difficult to model in laboratory animals [3]. Such obstacles of nature have been further complicated by nature's human interpreters who have imposed myriad discontinuous diagnostic categories [4] on disorders that are better conceptualized dimensionally, i.e. as quantitative deviations from health and as continuous symptoms spectra that cut across conventional disorder boundaries [5]. Even as neuroscience has flourished during the last several decades, such difficulties caused the neurobiological analysis of psychiatric disorders largely to founder.

Genetic analysis that associates phenotypes with specific variations in DNA sequence is a powerful tool for discovering the molecular underpinnings of heritable traits, including disease risk. The promise of genetics for psychiatry was long recognized based on the observations that schizophrenia, moods disorders, ASDs, and other such illnesses run in families and exhibit high heritabilities in twin studies [6,7]. Heritability is a measure of the influence of genes, compared with non-genetic influences such as environment, on the variation of a trait in populations. The concept of heritability has been misused in policy debates, for example with respect to the malleability of traits [8]. For the purposes of this discussion, the significant heritability of psychiatric disorders is important because it justifies investment in genetic analyses as a route to gaining biological insights.

Genetic information has particular utility for ferreting out causal mechanisms underlying a trait because a person's germ line DNA sequences are determined at the earliest stage of embryo formation. Thus, a DNA sequence variant that has been rigorously associated with a trait, such as schizophrenia risk, can be inferred to participate in causation. Because DNA sequences are determined *ab initio* with respect to each human being, this is a rare instance in which association can indicate causation. All other biological observations associated with a disease could play roles in causation, but could also be a result of the disease, represent an adaptation, or be confounded by prior treatment. Such assertions of scientific value come with caveats. As a result of stochastic errors in DNA replication that occur during the formation of the billions of somatic cells of the human body, germ-line DNA sequences may undergo mutation, including within neurons and glia of the developing brain where they could participate in disease pathogenesis [9]. Such genomic mosaicism contributes to some brain disorders, for example some focal epilepsies that result from somatic mutations that confer abnormal growth properties on affected cell lineages. That said, the familial transmission and high heritabilities of common neuropsychiatric disorders means that notwithstanding potentially pathogenic roles for somatic mosaicism, epigenetic modification, and other biological processes that influence genome structure and gene expression, germ line genomic sequences explain much of variance for many psychiatric disorders. A second caveat is that, the causal influence of a disease-associated DNA sequence variant on the phenotype may not always be direct or straightforward. For example, in an early genetic study of lung cancer risk, the most prominent risk associated genomic locus (i.e. place in the genome) was linked to genes that encode subunits of the nicotinic acetylcholine receptor—thus influencing smoking behaviour rather than more proximate causes of oncogenesis [10].

Given the promise of genetics to jump start research on psychiatric disease mechanisms, significant efforts were made in the 1990s and early 2000s with then available methodologies, most notably linkage studies, which had proven successful in identifying causative genes in rare monogenic disorders. The failure of linkage studies for psychiatric disorders indicates that the core assumption of this approach does not obtain for these phenotypes; it was therefore possible to reject the hypothesis that one or a few DNA sequence variants (alleles) of high penetrance was responsible for disease within families with multiple affected individuals. It became clear that risk for common psychiatric disorders was polygenic as Gottesman & Shields [11] predicted for schizophrenia. This means that the genetic component of disease risk results from the additive effects of many alleles of small effect, with none being necessary or sufficient. In an individual, genetic loading for risk-associated alleles (as opposed to neutral or potential protective alleles) would act together with stochastic developmental effects (such as somatic mutagenesis or chance patterns of gene expression in the developing brain) and environmental risk factors to produce illness. The predictions of Gottesman & Shields [11] followed from their observation that schizophrenia was transmitted within families in a non-Mendelian fashion (i.e. not following patterns expected for Mendelian dominant or recessive genes) and also from the failure of psychiatric disorders to segregate as uniform symptom complexes (thus undercutting the modern DSM classification before it was written). Another, methodologically far weaker approach than linkage, biological candidate gene studies and candidate gene-by-environment studies were also widely attempted in the 1990s—and unfortunately continue to this day despite stark failure, [12]. These approaches, which presuppose prior knowledge about causation to generate candidates, failed to recognize and correct for multiple testing procedures, and were vastly underpowered given what should have been obvious, the low penetrance of common alleles that contribute risk of psychiatric disorders. Indeed, a critical implication of polygenicity is the need for very large sample sizes in genetic studies given the need to identify alleles of low penetrance against the very noisy background of normal human sequence variation, comprised of tens of millions of sequence differences. A low penetrance risk allele for schizophrenia or depression will have no observable effect on the phenotype of interest except when the individual has an adequate number of other risk alleles. Thus most individuals with any given risk allele will not have the disorder, and depending on allele frequency in the population, most people with the disorder will not have any particular risk allele. Well conducted association studies are therefore powered to test the hypothesis that any given allele contributes to disease against a very large number of different genetic backgrounds. Studies of such large cohorts were simply not possible given the technology that existed prior to the Human Genome Project.

## 2. Genomic technology rescues genetics—but leaves neurobiology in peril

As has often been the case in the history of science, new tools can unleash scientific progress by making possible seminal new observations that engender new hypotheses [13]. For example, without improvements in the grinding of lenses, Galileo would not have been able to construct his telescopes that in turn, permitted him to observe the four brightest moons of Jupiter and thus convincingly alter our conception of the solar system. In the case of human genetics, the development of powerful new genomic and computational tools motivated by the Human Genome Project and related efforts made it possible to conduct unbiased, large-scale genetic studies of diverse human phenotypes. Unlike biological hypothesis-based candidate gene studies, modern unbiased approaches are based on finding association of traits with genetic markers or DNA sequences across the whole genome (or sometimes the whole exome, the protein coding regions of the genome) in a manner that is agnostic to biological hypotheses—a boon to psychiatry given its prior lack of knowledge of disease mechanism. Inexpensive DNA microarrays (‘gene chips’) made possible genome-wide association studies (GWAS) involving very large case-control cohorts to identify common disease-associated variants. Massively parallel DNA sequencing technology (often called Next Generation sequencing) vastly increased throughput and accuracy while diminishing costs by many thousand-fold. This has made possible well powered studies that have begun associate rare alleles ascertained by DNA sequencing with neuropsychiatric and other phenotypes [14]. Since analysis of genetically complex human phenotypes, such as neuropsychiatric disorders requires sample sizes beyond the reach of individual laboratories, the technological revolution in genomic could facilitate widespread success only with a change in the organization of human genetics, the formation of large international consortia that have demonstrated their value by successfully amassing large samples, typically many tens of thousands of individuals, and identifying DNA sequence variation associated with diverse phenotypes, ranging from anthropometric traits such as height [15] to risk of schizophrenia [16].

The information now emerging from unbiased, large-scale genetic studies of neuropsychiatric disorders is yielding initial clues to their biological underpinnings. That said, there remains a long and difficult road to travel if we are to understand disease mechanisms and advance therapeutics. Even assuming great success in the coming years in identifying a large fraction of the risk alleles that contribute to psychiatric disorders, and fine mapping loci identified by GWAS to identify causative variation—no mean feat—there is no guarantee that the resulting information will yield the desired result of understanding pathogenesis and giving birth to biomarkers, new treatments, and preventive interventions. This is because there is no clear paradigm, no map, no playbook, for follow-up biological studies of polygenic human brain disease.

The successful and widely used approaches by which biologists have studied gene function, most commonly the introduction of a single penetrant gene into a cell line or transgenic mouse, are well calibrated to identify the main effects of that gene against a single uniform genetic background of the chosen cell line or inbred mouse strain. How do we study a vast number of modestly penetrant alleles that alter human cognition and behaviour? We should not expect any isolated risk allele associated with psychiatric disorders to yield a meaningful phenotype in a mouse. Not only is the penetrance of almost all disease associated alleles low—the case not only for all common risk alleles, but also for most protein altering rare alleles—but genetic background matters greatly. Schizophrenia or depression require loading of many risk alleles that each nudge brain development or brain function toward illness. Given 80–90 million years of evolutionary distance from our last common ancestor with rodents, given the vastly different selective environments in which rodents and primate evolved, and given the empirical documentation of very poor conservation of genomic regions involved in regulating gene expression, we should not rely on mouse models to understand processes of disease causation that flow from polygenic risk. Given the large and punishing burden of these disorders on individuals, families, and societies, and a half-century in which both the efficacy of pharmacologic treatments and the range of symptoms treated has failed to progress significantly [17], it is incumbent on the field to embrace rather than avoid the experimental and conceptual challenges posed by the polygenicity and heterogeneity of psychiatric disorders, no matter how difficult, but to do so without falling prey to the kinds of intellectually lethal shortcuts that characterized the era of candidate gene studies.

## 3. What kind of evidence warrants biological follow-up studies of genetic associations?

Even before thinking about a new map with which to navigate polygenic *terra incognita*, we must address the question of what alleles and what genes can be considered well enough validated to warrant the investment in biological follow-up studies. At this point in history, the successful approach of basic science, which is to test biological hypotheses based on knowledge and intuition, represents a moral hazard for studies to follow up on genetics. What matters instead is the quality of the design and statistical power of the underlying genetic studies and the rigour of the analyses. Psychiatric genetics has learned hard lessons over the past decades that absent strong statistical evidence for association of an allele with the selected phenotype, no degree of biological plausibility in the mind of an investigator (which in this case should be reconstrued as no more than bias), warrants biological follow-up studies. Given the costs, human effort, and alternatives foregone, successful exploitation of genetic information about psychiatric phenotypes will require shared high standards by both the genetics and neurobiology communities for significance of associations. Sample size is the critical determinant of whether a study has the power to detect associations with the degree of certainty that would warrant follow-up [18]. For case-control association studies in neuropsychiatric genetics, designs must take into account allele frequency (i.e. whether the goal is to detect common or rare disease-associated variants), the likely effect size of disease-associated variants, and the number of independent tests being performed (which on a typical microarray used for GWAS is typically about 1 million.) To illustrate the role of sample size in psychiatric genetics, it is useful to use schizophrenia as an example. An international consortium performing genome wide (common variant) association in schizophrenia could not find significant associations until it reached nearly 10 000 cases and 12 000 controls [19]. By 2014, this consortium, by then enlarged, had performed GWAS on nearly 37 000 cases and a far larger number of controls and found 108 genome-wide significant loci associated with schizophrenia [16]. In ongoing not yet published studies involving yet larger cohorts (approximately 65 000 affected subjects and 85 000 control subjects), the number of significant associations has passed 250 (Stephan Ripke 2017, personal communication). Given the large number of common and rare risk variants in populations, individuals with schizophrenia (or any other psychiatric disease phenotype) will have genetic loading based on different combinations of risk alleles—a situation that probably contributes along with chance and environmental risk factors—to the well-known heterogeneity of psychiatric disorders in terms of such factors as symptoms, age of onset, severity and treatment responsiveness.

Despite such examples and the renewed focus of the biology and psychology communities on rigour and replicability, vastly underpowered candidate gene studies continue to be performed and published. Reliance by the neuroscience and psychology communities on false associations resulting from poorly designed and underpowered genetic studies have wasted significant resources and side-tracked the careers of many young investigators.

In addition to their statistical power, the unbiased designs of modern genetic association studies have contributed significantly to advancing biological investigation. In contrast to biological hypothesis-driven studies, genome-wide association studies can identify previously unsuspected biology. For example, a large GWAS study of schizophrenia [16] led to the identification of Complement Factor 4a (C4a) as a disease associated gene, with a very high degree of statistical confidence [20]. C4a, a component of the innate immune system, was not previously considered in the context of neuropsychiatric disease. This ‘new biology’ discovered through large-scale, unbiased genetics, has inspired a new focus on the role of synaptic strength and synaptic refinement mediated by complement proteins and microglia in schizophrenia.

Given the longstanding failure to advance therapeutics, unbiased approaches also free investigators from the purgatory of recycling a small number of hypotheses, many based on the initial molecular targets of serendipitously identified psychiatric drugs. Schildkraut & Kety [21], pioneers who initially formulated a biogenic amine hypothesis of depression, explicitly warned of the hazards of hypothesizing that disease mechanisms would represent the biological inverse of therapeutic drug action, a form of *post hoc ergo propter hoc* fallacy—as if pain were due to an aspirin deficiency. They wrote that despite the consistent ability of certain drugs to elevate or depress moods by increasing or decreasing monoamine neurotransmitter levels, what would be needed for insight into pathogenic mechanisms would be a ‘direct demonstration of the biochemical abnormality in the naturally occurring illness’, not an inference based on administration of pharmacologic agents. Further, they pointed out that even if such a biochemical abnormality were demonstrated in patients, it could still be an epiphenomenal downstream effect of some other aetiological factors, including environmental and experiential factors. Unfortunately, their advice was not well heeded. Unbiased, large-scale genetics can now provide the kind insights, grounded in the biology of affected individuals that Schildkraut and Kety saw as necessary to understand pathogenesis. Modern designs have the additional advantage—in contrast to the monoamine theory, which in fairness dates from a far earlier era—of identifying previously unsuspected mechanisms.

## 4. Genetic risk for psychiatric disorders is revealed to be fiendishly complex

The prediction of polygenicity made by Gottesman & Shields [11] for schizophrenia, extends to all psychiatric phenotypes that have been studied. It should be noted that there are rare cases in which symptoms of ASDs or of schizophrenia are associated with penetrant, damaging mutations in single genes or with a copy number variant (CNV), which produces a deletion, duplication, or more complex structural variation in a segment of the genome. Many of the single gene mutations associated with such cases occur *de novo* in the affected individual. Mutations that produce such severe neurodevelopmental phenotypes typically block production of an active protein from one of the human genes that do not tolerate haploinsufficiency—a situation in which healthy functioning requires that both copies of a gene are active. In essentially all cases of penetrant mutations or CNVs that increase risk of ASDs or schizophrenia, the most penetrant phenotype intellectual disability (Stefansson), and depending on the precise genes involved, other developmental abnormalities may occur, such as facial dysmorphology, cardiac defects, and epilepsy. In these cases, often described as syndromal ASDs or syndromal schizophrenia, the polygenic background still appears to play a role in phenotype determination, although more characterization is needed. Notwithstanding these rare cases, the rule for psychiatric phenotypes is extreme polygenicity—many alleles of small effect.

In the schizophrenia GWAS in which the Psychiatric Genomics Consortium identified 108 independent genome-wide significant loci for schizophrenia, the average odds ratio for associated alleles was 1.08 [16]. This translates into 8% increase in risk of a disorder with a population based rate of approximately 1%; thus, an average disease associated allele from this study would increase risk of schizophrenia from 1% to 1.08%. Schizophrenia is highly heritable, but the aggregate heritability is divided into many small additive contributions. The genetic loading of a person with schizophrenia represents a subset (above some unknown threshold) from the far larger number of risk alleles found in the population. For such extreme allelic heterogeneity to give rise to an identifiable, even if heterogeneous syndrome, it is generally hypothesized is that the effects of these many DNA sequence variants must ultimately converge on a far smaller number of biological processes, molecular pathways, and neural cell types.

A further complexity results from the pleiotropic effects of genes. In addition to significant allelic heterogeneity within a disorder, there is overlapping genetic risk across disorders [22]. Thus, schizophrenia and bipolar disorder share approximately 65% their common risk alleles [22], with still unknown combinations of risk alleles probably underlying phenotypes intermediate between schizophrenia and bipolar disorder that are often subsumed the term ‘schizoaffective disorder’. Genetic heterogeneity within disorder and sharing of risk across disorders are inconsistent with the narrow categorical definitions of disorders in the DSM-5 [4] Diverse patterns of shared and unshared genetic risk characterize many neuropsychiatric disorders [22].

It should also be noted that GWAS identifies places in the genome (loci) linked to causal variation and that further challenging steps are often required to identify the precise sequence difference that contribute to disease phenotypes. These and other steps will require much effort and ingenuity, but the genetics community largely knows how to go about them. In contrast, to this increasingly well-established map for genetic studies, approaches to understand the effects of polygenic influences on the biology underlying cognition, emotion, and neuropsychiatric disorders still represents a relatively trackless *terra incognita*.

## 5. From polygenic risk to psychiatric disease mechanisms: a new map must include non-reductionist strategies

Recent progress in neuroscience can scarcely be imagined without the application of reductionist approaches to experimentation. A classic example of success comes from investigation of memory mechanisms. It would not have been possible to gain significant scientific traction on mechanisms that underlie encoding and consolidation of memories if the initial experiments had been conducted in complex mammalian brains—even though the ultimate goal was to understand mammalian mechanisms. Instead, Kandel and colleagues began by investigating memory mechanisms in the sea slug *Aplysia californica*, an organism with an extremely simple nervous system. The greatly reduced complexity of the *Aplysia* nervous system facilitated a mechanistic analysis that yielded general principles relevant to the study of the far more complex brains [23]. The selection of a reduced experimental system must balance the simplicity required for successful application of available technology against the degree of complexity required achieve desired external generalizability. *Aplysia* fit this need. It is a simple living system, but not so simple as to preclude the application of experimental insights to investigation of mammalian memory mechanisms. Like any free-living organism, *Aplysia* needs to encode information about its world and to retrieve it in response to appropriate cues if it is to survive. The *Aplysia* nervous system is complex enough to model fundamental neural building blocks of memory that are conserved in evolution to the degree that they are generalizable to mammals in principle if not in every detail. In short, the utility of reductionist strategies depends on the degree to which the experimental system is well suited to answer scientific questions being asked in the short term, while informing longer term goals of the research programme.

Experimental paradigms in wide current use to investigate biological functions of genes, including disease associated genes, is to insert the gene into a clonal cell line or to generate a transgenic mouse in an inbred genetic background. Phenotypic differences resulting from the transgene are then identified in the modified cell line or mouse and compared with an appropriate control cell line or mouse that is genetically identical except for the inserted gene. Such approaches typically minimize confounding background ‘noise’ by selection of a uniform genetic background thus improving the likelihood that phenotypic differences from controls are due only to the experimentally introduced gene. Such reductionist methods have yielded important new information about the effects of protein truncating mutations in genes that have been associated with syndromal ASDs, including Neuroligin-3 [24] and Shank-3 [25]. These rigorously conducted studies have advanced understandings of the function of such disease-associated genes as Neuroligin-3 and Shank-3 and deficits that can result when they are made to be haploinsufficient. In addition these studies suggest new ideas relevant to the discovery of new therapeutic interventions. However, I have come to believe that such studies are better construed as basic science rather than as the production of disease models and would argue that this distinction is significant. Designation as an ‘animal model’ of disease invites misleading inferences that the animal reproduces important aspects of human disease mechanisms and further that it might be used to predict the efficacy of therapeutics. Designation as an experimental system for basic investigation invites a more exploratory posture with respect to disease biology and therapeutics.

In the past, it had been widely accepted that animals manipulated by genetic engineering, environmental perturbations, or breeding for disease-like traits could be validated as models of neuropsychiatric disorders based on three criteria (see [26]). Face validity entails a judgement that the animal's phenotype captures important characteristics of the human disease. The criterion of predictive validity is said to be met when assays conducted in an animal successfully predicts treatment efficacy in patients. Construct validity is based on the use of genetic or environmental factors in the construction of the putative model that are associated with disease aetiology in humans. Nestler & Hyman [26] have been sceptical of the concepts of face and predictive validity. Face validity suffers from its frequent reliance on subjective judgements, but more importantly because it is not a form of validation at all. This criterion would validate phenocopies, i.e. animals with phenotypes similar to those of ill humans, but with different biological underpinnings. Examples of convergent evolution (e.g. that insects and birds both have wings) should serve as a potent warning against reliance on surface phenomenology. Similarly, the criterion of predictive validity does not demand mechanisms that might be shared between the human disease and the constructed animal, and can thus represent a chance phenomenon. As a historical matter, animal based assays such as the forced swim test, have not yielded any drugs for approved psychiatric disorders except those that recapitulate the mechanism of prototype drugs involved in development of the assay—and first identified by their effects in humans [17].

Based on the findings now emerging from unbiased, large-scale genetic studies, I have grown pessimistic about the concept of construct validity, even with respect to the more penetrant mutations associated with syndromal ASDs and syndromal schizophrenia [3]. As is well known, the use of mice and rats as translational models of human neuropsychiatric disorders is severely limited by significant differences in neural cells types and the structure and function of neural circuits, most notably, but not limited to prefrontal cortex and its projections, which play critical roles in schizophrenia, mood and anxiety disorders, and the many other psychiatric disorders that affect cognitive control of thought, emotion, and behaviour. Such limitations in translatability are unsurprising given the 80–90 million years of evolutionary divergence since the last shared common ancestor of rodents and primates. More significantly, rodents and primates have evolved with vastly different selection pressures given the evolutionary niches they occupy. Rats and mice are nocturnal, modestly social, and specialized for olfaction; humans are diurnal, richly and complexly social, and highly visual. While these considerations militate against the acceptance of rodents as veridical models of disease, the do not argue against their use for diverse basic investigations including studies relevant to disease, especially given powerful technologies optimized for use in mice and rats such as optogenetics and *in vivo* microscopy.

Even if the neurobiology community foreswears the traditional concept of an animal model of disease in order to enhance interpretive discipline and reduce risks of self-deception, a severe challenge remains. In what experimental systems will it be possible to interrogate the causal influences exerted by diverse combinations of modestly penetrant alleles associated with psychiatric disorders? The challenges posed by polygenic background have recently been highlighted in elegant experiments conducted in mice. The use of inbred mouse strains as a background against which to study introduced transgenes is meant to minimize differences between experimental animals and controls. However, this practice also limits the generalizability of the results. A recent systematic study highlights this long-recognized problem. Sittig *et al*. [27] engineered mice to carry a severe mutation in one of two genes in which milder genetic variants have been found to be associated with neuropsychiatric disorders, including schizophrenia. When they bred the transgene into different inbred mouse lines (i.e. into different genetic backgrounds), they observed marked differences in the phenotypes, including the occasional disappearance of a trait or a change in its directionality compared with controls of the same strain. Sittig *et al*. [27] conclude that the phenotypic effects of rare deleterious mutations depend not only on the mutated gene, but also on its interactions with genetic background. This conclusion is fully consistent with observations in human patients in which the same penetrant single-gene mutation or CNV yields highly variable phenotypes [28].

Given that polygenic combinations of alleles underlie psychiatric disease risk in humans, and that noncoding regions of the human genome, which are poorly conserved across evolution, contain most of the common disease-associated loci, the already distant possibility of a genetic mouse model of schizophrenia or a mood disorder fades to impossibility. Moreover, the common and rare variants that contribute to risk of common psychiatric disorders have low penetrance, i.e. contribute small additive increments of risk that can produce a disease phenotype only in the context of many other human risk alleles. This signifies that transgenic mice that have constructed with common psychiatric disease risk variants cannot be expected to produce a disease-relevant phenotype, and assertions to the contrary are misleading.

## 6. Investigating *terra incognita*

It is important to state that there are currently no ideal experimental approaches to study polygenic human brain disease, at least without still undreamt-of technologies to advance human experimental biology. What is needed, if we are to take polygenic risk seriously rather than retreating to the basic investigation of mutations that cause rare monogenic disorders, are experimental systems that permit the interrogation and experimental manipulation of many human genomes (i.e. many different permutations that contain diverse risk and non-risk alleles). Successful study of many different genomes under different conditions will require inexpensive high throughput experimental systems and assays in order to achieve adequate statistical power to in the face of heterogeneity and irreducible experimental variability. Perhaps the most promising initial approaches are based on human cellular models generated by reprogramming (e.g. stem cell technology) and genome engineering, technologies that have emerged and matured only in the last decade [29]. Here I sketch variations on this theme that are still in early stages of development, recognizing that early technologies may not live up to their promise, and that even the most advanced three dimensional cellular models will not recapitulate human cognition or behaviour. Thus, while human cellular models will probably prove central to the interrogation of polygenic disease mechanisms, they will need to be complemented by basic animal research and by advances in human experimental biology.

The ability to reprogramme readily available human cells such as fibroblasts, either into pluripotent cells that can then be coaxed into any cellular phenotype [29], or directly into neurons, has made it possible to grow human neurons and glial cells with diverse human genetic background *in vitro*. In many current large-scale genetic studies, it is now an option to ask consenting patients for skin biopsies or extra blood samples out of which to produce pluripotent cell lines. There have been early reports that have compared neurons derived from three or four individuals with schizophrenia or bipolar disorder with healthy control subjects with claims of significant phenotypic differences. Given the heterogeneity of these conditions, it is extremely risky to draw conclusions from such small samples [18]. However, better designed and better powered studies are not far off. As the relevant technologies advance, high throughput methods of comparing cells derived from many individuals will become increasingly feasible. An important goal is to able to reprogramme, perturb, or otherwise probe cells from many individuals including those with disease phenotypes, different degrees of polygenic risk, or rare penetrant mutations under near-identical conditions. Gene variants can be added to or subtracted from the genomes of any cells (and thus from any human genetic background) using genome engineering technologies such as CRISPR-Cas9.

Given a focus on brain and brain disorders, there are additional complexities. There likely several thousand distinct cell types in the human brain; determination of their identities based on such factors as their stereotypic locations, morphologies and transcriptomes is a matter of intense current effort. At present it is only possible to generate a small minority of neural cell types for *in vitro* for experimentation; however, the number will grow. Help in studying neuropsychiatric disorders will arrive from the intersection of current efforts to generate a cell census of the brain (including a transcriptome of each cell type) and genetic studies. It will be possible using the resulting databases to identify those cell types that express a significant number of the genes associated with particular neuropsychiatric disorders.

The flat, two-dimensional cultures of individual human neural cell types described above have the advantage of relative simplicity and accessibility for manipulation. Although the initial actions of genes occur within cells, the symptoms and impairments of neuropsychiatric disorders do not reflect only cell-autonomous processes. There is much evidence for alterations in the structure and function of synaptic connections of the brain, making it critical to develop experimental systems in which multiple cell types can be elaborated and permitted to form synapses. Diverse approaches have therefore been taken to the production of so-called three-dimensional cultures. When such cultures are patterned to produce a limited number of neural cell types, they are often described as neural spheroids [30]. When permitted to develop over long periods and to develop a significant diversity of neural cell types they are called brain organoids [31]. Human brain organoids that have been grown for 8 months have been shown by electron microscopy and neurophysiology to develop synaptic spines and mature synapses. Physiological examination shows that spontaneous activity occurs between neurons in such organoids [32]. Both spheroids and organoids can be made from any available genetic background and from cell lines that have undergone genome engineering to introduce or edit out genes of interest, including genes associated with disease.

Both two and three dimensional human cellular models are experimental systems of reduced complexity, but they have the important property of permitting the study of neurons, glia and synapses with highly diverse genetic backgrounds derived from people who are well or who have a disorder under study. It is also proving possible to study cells from many individuals in parallel to maximize comparability of experimental conditions. However, it one has the goal of understanding the effects of disease risk or full-blown illness on brain circuits, cognition, and behaviour, then cellular models, while informative, will no longer suffice. Animals are critical for many basic science questions, including questions related to disease mechanisms, but given the polygenic nature of risk for all common neuropsychiatric disorders, it is an open question to what degree animals can be useful in translational science [33,34]. One promising avenue, albeit one that uses animals as a living incubator rather than as an independent experimental system is to transplant human neural progenitors derived from pluripotent cell lines into animal brains [35]. These cell lines can be made from disease affected individuals, diverse genetic backgrounds, and can also be engineered to express reporter genes for easy identification. In addition to cellular systems and experiments in animals, increased attention to human biology will pay important scientific dividends.

## 7. Summary

Here I have described possible causes of the failure to advance understandings of neuropsychiatric disease mechanisms and therapeutics over many decades. I believe that unbiased, large-scale genetics may be the most effective source of molecular clues that we will ever possess. Given the highly polygenic basis of genetic risk for neuropsychiatric disorders, I would argue that such genetic studies be prosecuted to the point of diminishing biological returns, a point at which the convergence of genetic information of molecular pathways, neural cell types, and biological processes should be adequately clear to be scientifically actionable. Discovery of an unsuspected role for complement factor 4a in schizophrenia is an early demonstration of the power of unbiased genetics at scale to reveal new biological insights. I have also reflected on the kinds of experimental systems that will be needed to interpret the functional consequences of polygenic risk. The current approach of generating transgenic mouse lines cannot answer the needs of interrogating phenotypes with a polygenic basis. Despite the strong constraints of operating within an entrenched paradigm [36], the unmet needs of patients should motivate a movement toward experimental systems that will make good use of the information now emerging from successful genetic analyses.

## Data accessibility

This article has no additional data.
